# Mammary Analogue Secretory Carcinoma of Submandibular gland

**DOI:** 10.22038/IJORL.2022.59803.3062

**Published:** 2022-05

**Authors:** Rupa Mehta, Sharmistha Chakravarty, Nitin M Nagarkar, Ashish K Gupta, Amit Banjare

**Affiliations:** 1 *Department of ENT and Head Neck Surgery, All India Institute of Medical Sciences, Raipur- 492099, Chhattisgarh, India.*; 2 *Department of Pathology, All India Institute of Medical Sciences (AIIMS), Raipur- 492099, Chhattisgarh, India.*

**Keywords:** Lymphatic cyst, Mammary Analogue Secretory Carcinoma, Neck dissection, Submandibular gland

## Abstract

**Introduction:**

Mammary Analogue Secretory Carcinoma of salivary glands (MASC) is a low-grade carcinoma of salivary glands of the head-neck region. It bears histological resemblance to Secretory Carcinoma of the breast and Acinic Cell Carcinoma (ACC) of the parotid gland. Its clinical behaviour and aggressiveness vary amongst individuals and experience in MASC of the submandibular gland are limited.

**Case Report:**

We report a 16-year-old female with binary neck swelling in the submandibular region. The hard swelling in the submandibular region was a MASC and the soft cystic mass was a synchronous congenital lymphatic cyst in the neck. We report two unusual features, an extremely rare involvement of MASC of submandibular salivary gland and the presence of a congenital lymphatic cyst in the area adjacent to the main tumour mass. Treatment was done by surgical excision of both the neck masses in-toto and ipsilateral selective neck dissection (Level I-IV).

**Conclusions:**

While MASC's histological pattern has been described in previous studies, its clinical picture is rarely documented. This report aims to shed light on the clinical presentation of this under-diagnosed entity and the aggressive management protocol required during preoperative workup, intraoperative disease clearance and post-operative follow up of such patients. MASC of the submandibular salivary gland is an uncommon cause of neck swelling in the adolescent age group, but due to its occasional aggressive nature, should be borne in mind as a possible differential diagnosis of salivary gland tumours.

## Introduction

Mammary Analogue Secretory Carcinoma of salivary glands (MASC) is a rare entity with barely 90 cases reported in world literature till 2014 and has been documented in the 4^th^ edition of WHO classification of head and neck tumours(2017) (1,2). 

MASCs are most commonly located in the parotid gland but can appear in other major or minor salivary glands. We report a case of rare and uncommon presentation of MASC of the submandibular salivary gland along with a lymphatic cyst of the neck in a 16yr-old female patient. The patient presented with two discrete neck swellings, one was a solid, firm to hard mass in the left submandibular region, and the other was a tense, cystic swelling adjacent to the first mass beneath the angle of the mandible. The two neck swellings were present as left-sided neck masses and hence differential diagnosis was with more frequent pathological entities.

MASC of salivary glands is more commonly reported in the parotid gland, though rare documentation in minor salivary glands of the oral cavity does exist (2). The purpose of reporting this case of MASC is because of its two unusual findings, namely unusual involvement of the submandibular salivary gland as a primary site and association of a lymphatic cyst adjacent to the tumour mass which has not been previously reported in the literature. This case report on MASC of the submandibular salivary gland describes the cytological, histopathological, radiological and clinical presentation of this neoplasm.

## Case Report

A 16-year-old female presented with complaints of insidious onset, gradually progressive left sided neck swelling for 10 years. Another swelling developed as a painless lump in the left submandibular region for 5 months, which was gradually increasing in size. There was no associated pain, difficulty in swallowing, difficulty in breathing, fever, weight loss, voice change, dental pain or any upper respiratory tract infection.

 There was no history of previous trauma, surgery or radiation exposure. She had no prior medical or surgical illness and took no medications for any major disease. 

**Fig 1:**
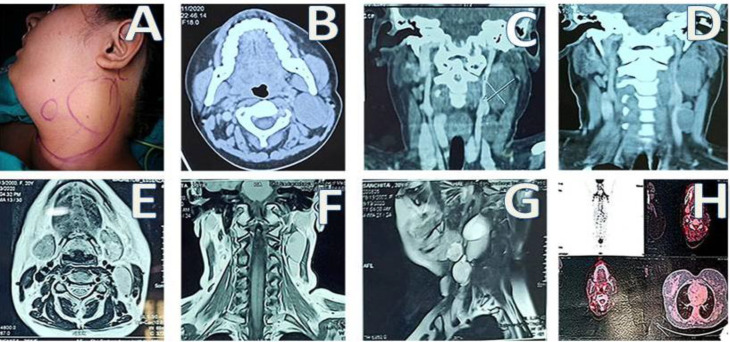
A: Two discrete swellings in the neck; B, C, D: CECT neck showed iso to hypodense lesions measuring 3.3x2.7x5.9cm at level II and III on the left side, E, F, G: MRI neck, H: 18F-FDG whole-body PET CT scan

On local examination, there were two swellings on the left side of the neck as shown in Fig. 1- A

1. Left level IB- 1.5x2 cm size swelling in the submandibular region which was firm, mobile, non-tender, no local rise of temperature, bi-manually palpable, non-ballotable and overlying skin normal and pinchable.
2. Left level II- 3X4 cm size cystic swelling which was fluctuant, non-tender, mobile, bi-manually non-palpable, no local rise of temperature and skin normal and pinchable.

Peripheral blood smear and all routine investigations were within normal limits, except ESR, which was 170mm at 1 hr.

Specific investigations included USG guided FNA, CECT neck, MRI of the neck and PET CT scan**. **

USG guided FNA was performed separately for the two neck swellings. The hard submandibular mass was rendered as MILAN CATEGORY V lesion, suspicious of malignancy with a possibility of a) epithelial myoepithelial carcinoma and b) pleomorphic adenoma. The second neck swelling at left level II showed benign cystic lesion with a possibility of 1) branchial cyst and 2) old hematoma. 

CECT neck(Fig. 1-B, C, D) was done from the hard palate up to D4 vertebra and showed three discrete lesions. Left submandibular region had a well-defined hypodense exophytic lesion, probably tumour mass, arising from the posteroinferior aspect of the left submandibular gland. The lesion was hypoenhancing on post-contrast studies with no evidence of calcification. USG correlation revealed homogenously hypoechoic with few hyperechoic spots without posterior acoustic shadowing. 

A second lesion (lymphatic cyst)was noted which was well-defined, elongated, lobulated extending lateral to carotid sheath and deep to sternocleidomastoid muscle at the level of cervical node station II and III on left side. Another well-defined lesion was noted in left level III of the neck, measuring 2.4X1.5X2.4cm. 

MRI neck (Fig.1- E, F, G) showed lobulated, inter-communicating cystic lesions in the left paraspinal prevertebral region deep to the left sternocleidomastoid muscle. Larger lesion measuring 5.5X3.5X2.3cm with a small inferior component of size 2.5X2.0X1.5cm. – possibly branchial cleft cyst. Focal STAIR hyperintense/ heterogenous T2 hyperintense lesion of size 1.8X1.7X1.9cm was noted adjacent to the left submandibular gland.18F-FDG PET CT scan (Fig.1- H) showed both FDG avid and non-FDG avid lesions. 

FDG avid well-defined hypodense soft tissue density lesion measuring approx 1.7X1.8X2.2cm (SUV max 7.2 vs 5.9 in delayed images), arising from the posteroinferior aspect of the left submandibular gland- likely neoplastic aetiology.

Non-FDG avid well defined soft tissue density lesion measuring approx 3.6X2.9cm noted at the level II and III lymph nodal stations deep to the left sternocleidomastoid muscle. Another lesion of similar morphology was noted inferiorly measuring 1.8X1.4cm at left level IV lymph nodal station- likely necrotic lymph nodes. FDG avid right level II (SUV max 6.3) and left level III cervical lymph nodes were also noted. 

Hence, radiological investigations revealed three swellings, one arising from the left submandibular gland, the second was a lymphatic/ branchial cyst at level II of the left side of the neck and the third one was a level III/IV lymph node.

**Fig 2:**
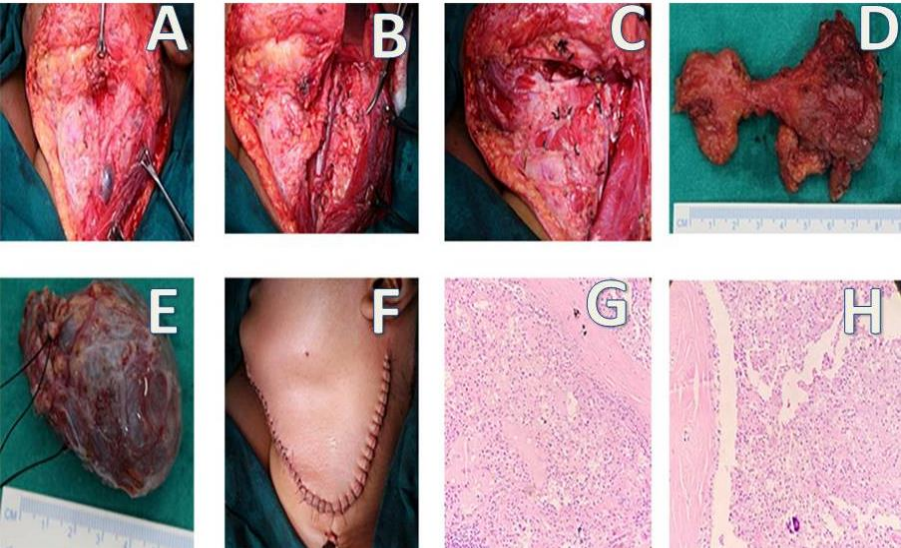
A-F: Intraoperative findings showing subplatysmal flap elevation and removal of the submandibular gland and lymphatic cyst with supra-omohyoid neck dissection, G, H: H and E staining: Magnification at 400 X, 200X

Based on the above findings, surgical planning was done and the patient underwent Left-sided submandibular gland excision with left Extended Supraomohyoid(Level I-IV) Neck Dissection with left sided branchial cyst excision (Fig. 2: A-F).

Intraoperatively, a horizontal upper neck skin incision was made on the left side from mastoid tip to mentum curving inferiorly at the level of the hyoid bone. The subplatysmal flap was elevated superiorly up to the mandible and inferiorly up to the inferior belly of omohyoid. Level IA was dissected between the bilateral anterior bellies of digastric muscle. Left level IB revealed a firm to hard mass infiltrating laterally up to the sternoleidomastoid muscle, inferomedially up to the posterior belly of digastric muscle and anteroinferiorly up to the superiorly belly of omohyoid muscle. The submandibular gland was excised and lingual and hypoglossal nerves were identified and preserved. Left level II cystic swelling of size approx. 5x6 cm was identified and removed ( superiorly extending up to skull base and inferiorly up to greater horn of hyoid). The Spinal accessory nerve was identified and preserved. Left level II lymph node with fibrofatty tissue was identified and sent for a frozen section which revealed reactive lymph nodes with cystic degeneration. As planned preoperatively, an ipsilateral selective (Level I-IV) neck dissection was performed and all the surgical specimens were labelled and sent for histopathology.

A final pathology (Fig. 2: G, H) showed a well-differentiated 7x3x2 cm tumour of the submandibular gland strongly suggestive of Mammary Analogue Carcinoma of Salivary Gland with no extra parenchymal extension. Immunohistochemistry tests revealed that the tumour epithelial cells were positive for Gross Cystic Disease Fluid Protein (GCDFP-15) and Mammaglobin (MAM) and secretions were highlighted by Mucicarmine and Periodic Acid Schiff (PAS) stains. The histopathology of the second specimen of grey-white tissue of size 6.5x4x2cm was suggestive of a lymphatic cyst. The patient recovered well in the post-operative period and has been on regular follow up till date. 

## Discussion

Binary neck swellings in the submandibular region can be solid or cystic. Swellings in the neck with a cystic consistency can include a wide range of congenital and acquired lesions like branchial cleft cysts, lymphatic cysts, epidermoid cysts, laryngocoele, ranulas, carotid body tumours, etc. Solid neck swellings in submandibular regions include sialolithiasis, submandibular gland benign and malignant tumours, tubercular lymphadenitis, metastatic lymph node in occult thyroid carcinoma, neurogenic tumours, etc. We report a rare presentation of Mammary Analogue Secretory Carcinoma of the Salivary gland, rarer because it involved the submandibular salivary gland and the first reported case of MASC with a synchronous ipsilateral lymphatic cyst in the neck.MASC of salivary glands is a newly diagnosed pathological entity and histological studies have been reported, but the clinical picture in terms of presentation, radiological findings and surgical intervention as treatment modalities have been rarely highlighted in the medical literature. Mammary Analogue Secretory Carcinoma of salivary glands (MASC) is a newly diagnosed neoplasm that shares a histological and molecular resemblance to secretory carcinoma of the breast. It is known to harbour a specific genetic alteration defined by the t(12;15)(q13;q25) translocation, which results in the fusion of the ETV6 gene from chromosome 12 and the NTRK3 gene from chromosome 15 finally leading to the formation of a chimeric protein “tyrosine kinase”. Secretory carcinoma of salivary glands was previously confused with other salivary gland tumours and the main differential diagnosis were mainly Acinic cell carcinoma (ACC), mucoepidermoid carcinoma and cribriform cystadeno-carcinoma of the salivary gland. Of these, major histo-morphological similarities were with acinic cell carcinoma leading to a diagnostic dilemma. 

With the advent of newer diagnostic modalities like Immunohistochemistry and Fluorescent In-Situ Hybridisation techniques (FISH), this new entity of salivary gland neoplasm came into existence in 2010 as published by Skalova et al in a series of 16 cases (3). The authors christened it as Mammary Analogue Secretory Carcinoma of salivary glands (MASC). In the study, 16 cases of previously diagnosed acinic cell carcinoma (ACC) or adenocarcinoma not otherwise specified (ADC-NOS) were reviewed. In the paper, the predominant location was in the parotid salivary gland (13/16 cases) and three cases originated in the minor salivary glands of the oral cavity (buccal mucosa, upper lip and palate). Hence the tumour presented in our case is a rare documented case of MASC of the submandibular salivary gland and first-ever reported case of Mammary analogue secretory carcinoma of the submandibular salivary gland along with a lymphatic cyst in the neck.

Certain oncologic entities like adenoid cystic carcinoma, mucoepidermoid carcinoma, acinic cell carcinoma and pleomorphic adenoma occur in both mammary glands and salivary glands in the neck. This can be explained based on common embryological (ectodermal) origin and histological (exocrine) organization of the mammary and salivary glands. 

The majority (85–90% of cases) of Acinic cell carcinomas (ACC) have been documented in the parotid (4–8). This can be explained based on the predominance of serous acinar cells in the parotid compared to other salivary glands and parotid being the largest salivary gland, there is an abundant pool of acinar cells. Only about 5 to 10% of AciCCs arise outside from non-parotid sites given their paucity of normal serous acini (9-11).

The clinical scenario of this tumour and the true picture of the incidence of MASC is unclear. The histological resemblances to other pathological tissues have led to inappropriate classification in the past. In the study by Luk et al, 9 MASC were reported after a meticulous assessment of 190 malignant salivary gland tumours (~4.5%), whereas in an almost identical study by Majewska et al. 7 MASC were highlighted amongst a pool of 183 cases (~4%) (12,13). Gender distribution of MASC shows almost equal prevalence in males and females with a slight predominance in males. It commonly has shown to be a low-grade tumour with an indolent course, but cases have been reported with aggressive clinical behaviour with poor outcomes that can vary amongst individuals (14). MASC (mammary analogue secretory carcinoma of the salivary glands) is a solitary, well-circumscribed and encapsulated tumour. Histopathologic features, immuno-histochemistry and epidemiological aspects of MASC were outlined in various studies (13-17). Histological studies have described microcystic, macrocystic, tubular, papillary and solid structures, and commonly demonstrated small to medium-sized cells with eosinophilic and vacuolated cytoplasm and small nuclei. Immunohistochemistry studies have enabled confirmation of the diagnosis of MASC and establish a distinct entity of this subgroup of malignancy. These tumours show strong mammaglobin, vimentin, and S-100protein positivity. It is essential to differentiate ACC from MASC. According to an interesting study by Griffith et al., both histological picture and clinical presentation with a description of the intraoperative findings in terms of consistency, adhesions and locoregional spread provide supportive aids to arrive at the diagnosis of MASC (11). Another intriguing aspect in the differentiation of ACC from MASC is the presence of zymogen vacuoles which is not present in MASC. Genetic studies offer an excellent tool of diagnosis including ETV6-NTRK3 gene fusion t(12;15)(p13;q25) translocation study via Fluorescent In Situ Hybridization (FISH) analysis. As technology advances, greater diagnostic aids are added to the kitty. New features like the irregularity of nuclear membrane in addition to the solid/cystic component of the tumour have been recently highlighted in a study by Gonzalez et al in 2018 (17). With the addition of nuclear membrane criteria, multiple cases were reestablished as MASC which were hitherto diagnosed as ACC (2,17,18,).

The diagnosis is further backed up by various imaging methods like ultrasonography of the neck and CECT/MRI scan of the neck which helps in preoperative assessment of tumour size, vascularity, lymph node involvement and proximity to major vessels. (17,18). Along with Fine needle aspiration studies, newer diagnostic tools like immunohistochemistry for mammaglobin, vimentin and S-100 can supplement the final diagnosis. Hormone assay mainly based on ER, PR and HER-2 receptor status has been used in selective studies (19). MASC is a low-grade carcinoma with an indolent course. Chances of metastasis and recurrence of disease after surgical excision depends on the tumour size at the time of presentation. Perineural and lymphovascular invasion is uncommon and hence prognosis remains favourable as per available medical literature (3,18). 

MASC, being a low-grade malignancy, small size tumours are managed by complete surgical excision of the tumour mass. For larger size tumours, excision with neck dissection, radiation and chemotherapy is recommended for invasive or metastatic disease. In our case, the patient had two discrete neck swellings on the left side with ipsilateral level III lymph node enlargement. Complete surgical excision of the submandibular mass and the lymphatic cyst was done along with ipsilateral selective (level I-IV) neck dissection. 

The patient is asymptomatic thereafter with no recurrence after six months and is on regular follow up every three months.

Currently, the rare presentation of this disease entity has led to inadequate guidelines regarding its management protocols. Gene therapy has shown challenging results in Mammary Analogue Secretory Carcinomatic targeted therapy. Drilon et al. described a case where a clinically significant response was noted by utilizing targeted therapy with the pan-Trk inhibitor entrectinib (Ignyta) (20). 

This drug mainly targets cells that are positive for NTRK1/2/3 fusions. The identification of the ETV6-NTRK3 gene fusion can lead to revised and upgraded tumour classification and act as a torchbearer for future studies that will establish insights in terms of management strategies of MASC.

## Conclusion

Our case presented with two neck swellings in the submandibular region of the left side with a probable differential diagnosis of malignant submandibular gland tumour with metastasis, tubercular lymphadenopathy or metastasis in occult thyroid carcinoma. Malignant tumours of submandibular glands are commonly mucoepidermoid and acinic cell carcinoma. The possibility of Mammary Analogue Secretory Carcinoma of salivary glands is seldom kept in mind during an approach to a submandibular region mass with a second cystic mass in close vicinity. The presence of a malignant mass along with a lymphatic cyst extending up to the skull base is quite intriguing in itself. MASC is rarely seen in the submandibular gland and the co-existence of MASC of submandibular gland with a lymphatic cyst has not been reported previously in the literature. Although relatively rare, we need to keep this entity in mind for the differential diagnosis of salivary gland tumours.

This report aims to shed light on the clinical presentation of this underdiagnosed entity and the aggressive management protocol required during preoperative workup, intraoperative disease clearance and post-operative follow up of such patients. We herein try to highlight the possibility of MASC as a differential diagnosis for a firm to hard neck swelling in the head and neck region and meticulous management is imperative for the management of this entity.
